# Intraspecific variation buffers projected climate change impacts on *Pinus contorta*

**DOI:** 10.1002/ece3.426

**Published:** 2013-01-17

**Authors:** Brian Oney, Björn Reineking, Gregory O'Neill, Juergen Kreyling

**Affiliations:** 1 Biogeographical Modelling, University of Bayreuth, BayCEERBayreuth, Germany; 2 Research, Innovation and Knowledgement Management Branch, BC Ministry of Forests and RangeVernon, British Columbia, Canada; 3 Biogeography, University of Bayreuth, BayCEERBayreuth, Germany

**Keywords:** Intraspecific diversity, lodgepole pine, niche modeling, North America, range shift, within-species variability

## Abstract

Species distribution modeling (SDM) is an important tool to assess the impact of global environmental change. Many species exhibit ecologically relevant intraspecific variation, and few studies have analyzed its relevance for SDM. Here, we compared three SDM techniques for the highly variable species *Pinus contorta*. First, applying a conventional SDM approach, we used MaxEnt to model the subject as a single species (species model), based on presence–absence observations. Second, we used MaxEnt to model each of the three most prevalent subspecies independently and combined their projected distributions (subspecies model). Finally, we used a universal growth transfer function (UTF), an approach to incorporate intraspecific variation utilizing provenance trial tree growth data. Different model approaches performed similarly when predicting current distributions. MaxEnt model discrimination was greater (AUC – species model: 0.94, subspecies model: 0.95, UTF: 0.89), but the UTF was better calibrated (slope and bias – species model: 1.31 and −0.58, subspecies model: 1.44 and −0.43, UTF: 1.01 and 0.04, respectively). Contrastingly, for future climatic conditions, projections of lodgepole pine habitat suitability diverged. In particular, when the species' intraspecific variability was acknowledged, the species was projected to better tolerate climatic change as related to suitable habitat without migration (subspecies model: 26% habitat loss or UTF: 24% habitat loss vs. species model: 60% habitat loss), and given unlimited migration may increase amount of suitable habitat (subspecies model: 8% habitat gain or UTF: 12% habitat gain vs. species model: 51% habitat loss) in the climatic period 2070–2100 (SRES A2 scenario, HADCM3). We conclude that models derived from within-species data produce different and better projections, and coincide with ecological theory. Furthermore, we conclude that intraspecific variation may buffer against adverse effects of climate change. A key future research challenge lies in assessing the extent to which species can utilize intraspecific variation under rapid environmental change.

## Introduction

Projections of climate change and the related impacts on species distributions suggest significant ecological disturbance, especially when considering species' range losses (e.g., Thomas et al. 2004; Urban et al. 2012). Modeling the future potential distribution of 1,350 European plant species under various greenhouse-gas emission scenarios indicates that more than half of these species could become vulnerable, endangered, critically endangered, or committed to extinction in the climatic period 2070–2100 if unable to disperse (Thuiller et al. 2005). Species distribution modeling (SDM) encompasses a broad range of techniques (Guisan and Zimmermann 2000), and applications ranging from paleobiology (Svenning et al. 2011) to spread and control of infectious vector-borne disease (Fischer et al. 2011). However, major uncertainties are associated with SDM, such as the realistic modeling of migration rates (Best et al. 2007; Nathan et al. 2011), biotic interactions (Araújo and Luoto 2007; Preston et al. 2008; Meier et al. 2010), for example, fecundity and competition (Clark et al. 2011), consideration of micro-climate (Dobrowski 2011; Hof et al. 2011; Suggitt et al. 2011) or climate extremes (Zimmermann et al. 2009), and persistence of ecosystem structure and reliance upon other predictions (Dormann 2007; Wiens et al. 2009). Nonetheless, successful retrospective predictions of shifts in bird population sizes demonstrate the value of species distribution models (Green et al. 2008). Currently, migration rates and pathways are added to the models, thereby increasing realism of the projected results while still being restricted by limited species-specific knowledge on potential migration rates (Fischer et al. 2011). Austin (2007) presented a general framework for SDM studies, such that ecological theory must concur with a data model and statistical model. The incorporation of variation within a species or closely related group of species has been recently addressed as an important challenge to improve SDM (Zimmermann et al. 2010). However, “species” is a taxonomic designation, and may not necessarily designate an ecologically homogeneous group of organisms, especially when intraspecific ecotypes occur. Experimental evidence suggests that conventional SDM cannot capture the climatic response of species by treating them as homogeneous units (Beierkuhnlein et al. 2011). However, O'Neill et al. (2008) present an interesting approach to account for intraspecific variation of growth using provenance trial data. Similarly, Benito Garzón et al. (2011) investigated future tree survival when considering population variation, and found that acknowledgment of this variability provides a more positive projected outlook into future climates when compared with conventional SDM approaches. Furthermore, Pearman et al. (2010) note a slight improvement of their models when incorporating within-species variation when compared with the traditional SDM method. Hamann and Wang (2006) developed an interesting community modeling approach to account for ecosystem level variability, which has been successfully applied to highly prevalent tree populations, including *Pinus contorta*, in order to consider future development of local forest stands under climate change scenarios (Gray and Hamann 2012) as well as past climates (Roberts and Hamann 2012). The “ecosystem-based” approach has several advantages over species models, one being the ability to model individual populations in a changing environment (Roberts and Hamann 2012). Within-species variation, according to the insurance hypothesis (Yachi and Loreau 1999), contributes to a species' ability to utilize various resources (Joshi et al. 2001; Kreyling et al. 2012a,b) and thereby adapt to ecological change (Davis et al. 2005; Nussey et al. 2005; Skelly et al. 2007).

*Pinus contorta* is a pyrophilic, widely distributed, outcrossing, wind-dispersed conifer species of high ecological (Lotan and Critchfield 1990) and economic importance (Krieger 2001; Karst 2010). Naturally and through human influence, the economically important subspecies *latifolia* has been advancing northward since the end of the last glacial maximum (MacDonald 1991; Fazekas and Yeh 2006) and recent observations hint toward a continued northward spread (Johnstone and Chapin 2003). *Pinus contorta* exhibits great ecological variation, and can be separated into three genetically and ecologically distinct subspecies: *contorta*, *murrayana* and *latifolia* (Lotan and Critchfield 1990), making it an ideal candidate to investigate the effects of intraspecific variability on SDM.

We argue that the integration of within-species variation is a necessary step when considering ecological theory in SDM. We investigated the effect of incorporating intraspecific variation on SDM performance and projections using range-wide, geo-referenced, subspecies information and provenance test data of *Pinus contorta*. Two approaches driven by intraspecific data were compared with conventional SDM, which allowed for robust comparison of our findings against a particular way to incorporate intraspecific variability. We expected a difference in modeling results between approaches that ignore intraspecific variation and those that incorporate intraspecific variation. Furthermore, given the different theoretical backgrounds of the modeling approaches, we expected substantial differences in performance between modeling approaches.

## Methods

### Climate data

The worldclim climate data (Hijmans et al. 2005) was used with a resolution of 2.5 arcminutes, which was reprojected to Albers Equal Area projection (resolution of 4 km^2^) with GRASS GIS (GRASS Development Team 2012). Climate variables were derived using the worldclim dataset. Climate variable derivation formulae (Table 1) were taken from two sources: those used in O'Neill et al. (2008), and additional bioclimatic variables from Hijmans et al. (2005). Present climate data (1950–2000) were used for model training and validation, and future climate simulations were used for SDM model projections (HadCM3, A2a emissions scenario; Nakićenović et al. 2000) for periods 2010–2040, 2040–2070, and 2070–2100. Considering current CO2 emissions as well as plausible future political and social developments, the use of A2 simulation data appears justified (Moss et al. 2010).

**Table 1:** Climate variables investigated across the ranges of *Pinus contorta* and subspecies

Climate variables	Unit	Source
Annual Heat/Moisture Index	*^°^*C/mm	Wang et al. (2006)
Summer Heat/Moisture Index	*^°^*C/mm	Wang et al. (2006
Mean Annual Temp.	*^°^*C	Wang et al. (2006)
Mean Warm Monthly Temp.	*^°^*C	Wang et al. (2006)
Mean Cold Monthly Temp.	*^°^*C	Wang et al. (2006)
Temp. Difference/Annual Range	*^°^*C	Wang et al. (2006)
Mean Annual Precip.	mm	Wang et al. (2006)
Mean Summer Precip.	mm	Wang et al. (2006)
Isothermality	*^°^*C/*^°^*C	Hijmans et al. (2005)
Mean Diurnal Range	*^°^*C	Hijmans et al. (2005)
Temp. Seasonality	*^°^*C	Hijmans et al. (2005)
Mean Temp. of Wettest Quarter	*^°^*C	Hijmans et al. (2005)
Mean Temp. of Driest Quarter	*^°^*C	Hijmans et al. (2005)
Mean Temp. of Warmest Quarter	*^°^*C	Hijmans et al. (2005)
Mean Temp. of Coldest Quarter	*^°^*C	Hijmans et al. (2005)
Precip. of Wettest Month	mm	Hijmans et al. (2005)
Precip. of Driest Month	mm	Hijmans et al. (2005)
Precip. Seasonality	mm	Hijmans et al. (2005)
Precip. of Wettest Quarter	mm	Hijmans et al. (2005)
Precip. of Driest Quarter	mm	Hijmans et al. (2005)
Precip. of Coldest Quarter	mm	Hijmans et al. (2005)
Precip. of Warmest Quarter	mm	Hijmans et al. (2005)
Mean Max. Temp. of Driest Quarter	*^°^*C	This study

### Occurrence data

**Figure 1:**
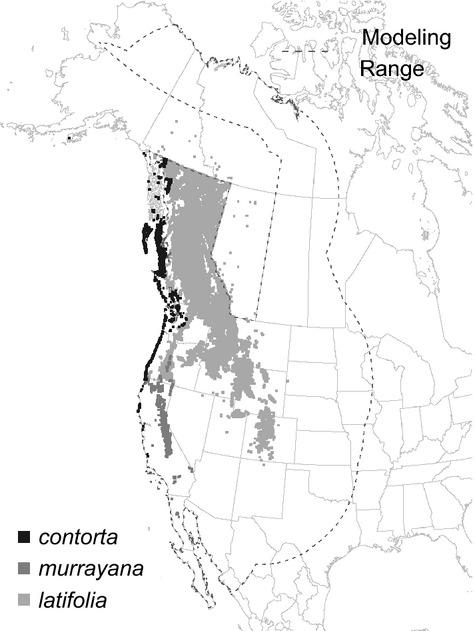
*Pinus contorta* subspecies distributions across its natural range. The number of observations documented to subspecies was low; therefore the nearest observation that was classified to subspecies was assigned that observations' subspecies classification. The dashed line outlines the model building buffer area i.e. model training and evaluation (see Supporting Information). Occurrence data were obtained mainly from the British Columbia Ministry of Forests and US Forest Service Forest Inventory and Analysis; the observational data cover most of the natural range. Within the modeling range, each raster cell was assigned either a presence or absence.

Occurrence data (Fig. 1) were obtained mainly from the Vegetation Resource Inventory of the British Columbia (BC) Forest Service, provided at a 1600-m grid and the US Forest Service, provided on a 10-km grid. Also, occurrence data were further supplemented with online resources such as herbaria, botanical gardens, and plant databases (see Supporting Information). Each dataset was examined extensively for outliers, relative to the most comprehensive *P. contorta* occurrence data from Little (1971). Unfortunately, most occurrences were not documented and classified to subspecies. In order to address this problem, occurrences without a subspecies classification were assigned the subspecies of their nearest spatial neighbor (see Supporting Information). The nearest neighbor analysis assigned the subspecies *contorta* to some observations that had elevations far outside its observed elevational range. Lotan and Critchfield (1990) describe *P. contorta* subspecies *contorta* as occurring mainly between sea-level and 610-m altitude. One of the *P. contorta* subspecies *contorta* provenances used in the Illingworth trial (O'Neill et al. 2008) was sampled at an elevation of 1266 m (sampled with the reprojected worldclim-SRTM elevation database); we used this value as the upper elevational limit of *P. contorta* subspecies *contorta*. These observations were in areas in the Pacific Northwest where subspecies *latifolia* and subspecies *contorta* introgress, and were therefore assigned to subspecies *latifolia*. Observations within a raster cell for each subspecies *contorta*: 2048, *murrayana*: 1449, *latifolia*: 42,342, and totaled: 45,785. The sum of the subspecies observations does not equal the total observations due to co-occurrence (see Supporting Information).

### Range designation

In order to avoid biased niche estimates arising from treating areas as absence that are climatically suitable but are not occupied because of geographic isolation, a buffer of 1,000 km around the occurrence dataset was calculated for the entire species as well as for each subspecies for model building and evaluation. Each model was trained on its own buffer (see Supporting Information), evaluated on probabilities within the whole species buffer (Fig. 1 – “Modeling Range”), and projected to the North American continent north of 23°N. *Pinus contorta* has yet to be documented to occur north of 65°N or in Saskatchewan, except in the southwest corner (Little 1971). We were unable to obtain a thorough dataset for Alberta, whose area was, except for presence data, omitted from the analyses. Areas lying within the buffer of 1000 km, but well outside the documented natural range of *P. contorta* were included as absence data. This meant that, within the buffer, areas north of 65°N, west of the Alberta-Saskatchewan border, or areas much further (>500 km) away from the edges of the observations were considered to be absence data. Areas of southern Alaska were omitted from analysis on the grounds that the sampling intensity is sparse in this region, and because two observations (Arctos Museum of the North, 2010) indicate possibly viable populations in south-central Alaska (see Supporting Information).

### Modeling approaches

Three SDM approaches were used to model distributions of *P. contorta*. MaxEnt (Phillips et al. 2006) was used (1) as a conventional SDM (Guisan and Zimmermann 2000; Wiens et al. 2009), the species observational data were modeled as if the species was ecotypically homogeneous, called hereafter the “species model”. In order to incorporate intraspecific variation, the most prevalent *P. contorta* subspecies (*contorta*, *murrayana*, and *latifolia*) were modeled; (2) as autonomous units, call hereafter the “subspecies model”. Finally, the “Universal Transfer Function” (UTF) from O'Neill et al. (2008) was used (3) to incorporate the observed variability in provenance trial tree growth.

#### Species model

Candidate climate variables were tested for collinearity with each other with Spearman's non- parametric correlation. Correlation among candidate climate variables was examined. Where pairs of variables were highly correlated (*ρ* > 0.7), a univariate generalized additive model (GAM) was fitted to the test data (see below) using each highly correlated variable. In order to obtain less correlated variables, the variable of each pair that yielded the greater AIC was omitted. MaxEnt (Phillips et al. 2006; Maximum Entropy, version 3.3a) was then used to model the current distribution with the full occurrence dataset. Austin (2007) discussed species response curves that coincide with ecological theory and we assumed a smooth response along a climatic gradient. Therefore, MaxEnt “feature” types linear, product and quadratic features were used (Elith et al. 2011), and the “samples-with-data” (SWD) input data format (see Appendix S4 in Elith et al. 2011). Otherwise, default settings were used. We use the term “absence data” to refer to the MaxEnt analogy “background samples”, and model absence data as background psamples (MaxEnt argument: environmentallayers).

#### Subspecies model

Each subspecies was treated as its own viable species, and was individually investigated for determinant climate variables. This was done using the same algorithm as for the “species model” approach. The resultant *N* subspecies model probabilities *P*_subsp,i_ at a given location *x* were then combined (Eq. 1) as in Pearman et al. (2010) to yield the occurrence probability of the species as a whole, *P*_tot_:


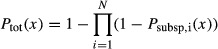
(1)

#### Universal transfer function approach

The Illingworth provenance trial (Illingworth 1978) began in the late 1960′s, taking seed from 140 provenances across most of the range of *P. contorta*, growing seeds in a nursery, and planting the 3-year-old seedlings across BC and Yukon Territory in 1974. An incomplete factorial design was used to test 60 populations at each of 60 test sites. We derived the UTF according to the same methods from O'Neill et al. (2008), using the Illingworth trial 35- year-old tree plot growth data (volume per hectare [V PH], m^3^/ha), a measure that combines height, diameter and survival. Using the original model structure, tree plot growth data were used to fit the UTF to the worldclim climate variables. The UTF projections were produced in two steps. First, at each test, site (*S*), individual provenance (*P*), mean cold month temperature (MCMT_P_) were fitted to population production (VPH_P_) using a unimodal Cauchy function to develop an individual transfer function for each test site:


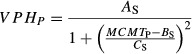
(2)

where *A*_S_, *B*_S_ and *C*_S_ are the fitted function parameters, yielding a transfer function per trial site S (*N* = 42, see Eq. 2; for background see Raymond and Lindgren (1990); Lindgren and Ying (2000)). Inter-site variation in the transfer functions, that is, the variation in the transfer function parameters *A*_S_, *B*_S_ and *C*_S_ was then modeled as a function of site climate, using mean annual temperature, mean coldest month temperature, and annual heat/moisture index (see O'Neill et al. 2008; for details). The resulting UTF (Eq. 2) predicts 35-year-old tree growth of a population from any climate growing in any climate (VPH_S_), and was used to predict current and future growth estimates. The growth estimates for each raster cell were then used to explain the observational data using logistic regression. The logistic regression model was then used to convert present and future growth estimates to occurrence probabilities.

### Model evaluation

Observations were randomly partitioned into two datasets, with 70% of the cells selected for training and the remaining 30% of the cells used for model evaluation, as is common in SDM studies (e.g., Thuiller et al. 2004; Fischer et al. 2011). The same set of cells was used for training and evaluating all models, that is, across all three approaches. Model performance was assessed by the area under the receiving operator characteristic curve (AUC), which measures discrimination, the Nagelkerke-Cox-Snell-Maddala-Magee *R*^2^–index, a general indicator of performance, and the logistic calibration curve, of which the slope indicates over- or underfitting (calibration) and the intercept indicates bias (see Reineking and Schröder 2006). A calibration curve slope greater than 1 (*α* > 1) indicates under-fitting/over-regularization, vice versa for *α* < 1, and bias indicates how well the numerical values of the actual and predicted probabilities correspond; that is, an intercept of less than 1 (*β* > 1) indicates that the predicted probabilities are too high and vice versa. The variable “importance” was calculated by randomizing (*n* = 10) each variable and then subtracting the mean change in AUC from the full model, and each AUC difference is divided by the sum of all AUC changes to derive the relative importance.

### Suitable area quantitation

In order to avoid the somewhat arbitrary procedure of threshold choice (Fielding and Bell 1997), the total area of habitat suitability *A_tot_* was quantitated by multiplying the predicted probability of occurrence *P*_*j*_ for each cell *j* with the area of the cell *Area_j_* and summing over the *M* analyzed cells (Eq. 3).


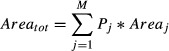
(3)

The AUC is a rank-based measure, which allowed us to quantitate the discriminative ability of the models, without choosing a threshold and creating a projected binary map of presences and absences. We refer to habitat suitability as the modeled probability of occurrence, which is important for predicting the success of seedlings, but also serves as reference for production potential, although adult trees have been observed to tolerate a wide range of conditions. The relative amounts of suitable habitat were quantitated with two dispersal scenarios: “no dispersal” and “full dispersal”. A full dispersal scenario is the optimistic extreme and assumes that the species can reach all habitats on the North American continent. The no dispersal scenario is the pessimistic extreme, and assumes that the species will not be able to migrate. These dispersal scenarios were used to bound and illustrate the range of possible future locations of the species' climate habitat, and to guide assisted migration efforts. Because we did not set a probability threshold to create a presence–absence map from the model projections, we took the occurrence data and quantitated the area of suitable habitat at each occurrence location. All data preparation, analyses, and visualization were conducted in R (R Development Core Team 2012) and GRASS GIS (GRASS Development Team 2012, version 6.4).

## Results

### Model performance

**Figure 2:**
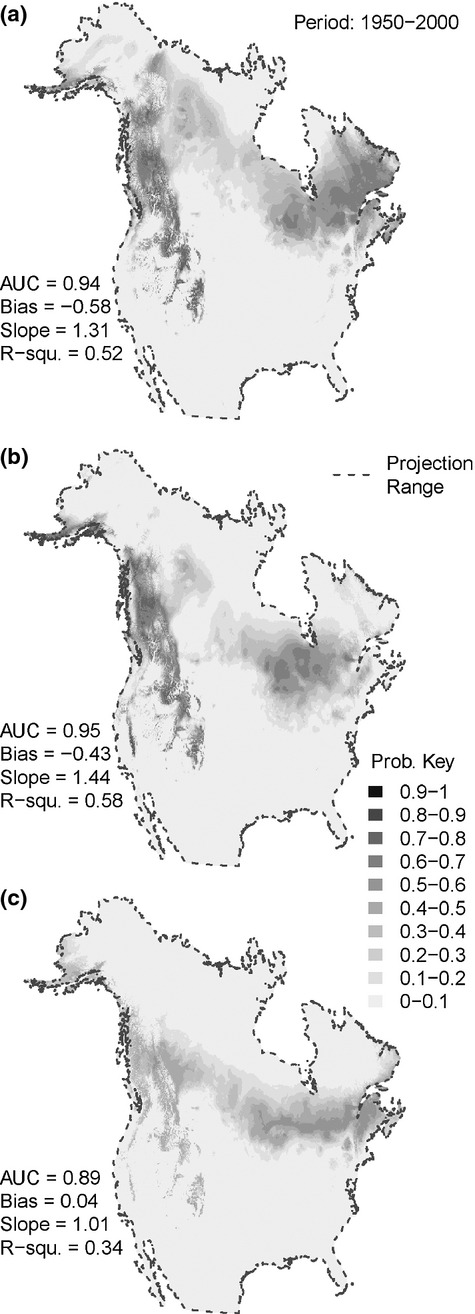
Occurrence probability distributions were modeled in the current climatic period (1950–2000) and the three models (a) species model (b) subspecies model and (c) universal transfer function (UTF) were projected onto North American climate data (Hijmans et al. 2005). The models were trained and evaluated on the modeling range (see Fig. 1) and projected to the projection range (dashed line), which corresponds to most of the North American continent. The MaxEnt models are highly discriminative, but the UTF is better calibrated.

For each MaxEnt model target, a separate set of climate variables was found to be best suited for describing the occurrence distributions, and the response curve characteristics give insight into the relationship between *P. contorta* distribution and climate (Table 2). The shapes of the curves representing the response of each subspecies provide more evidence that each subspecies should react differently to climatic changes. It is interesting to note the variety with which the models depict each subspecies, especially when comparing with the “species” model. In general, the models performed well. According to the *R*^2^ -index and AUC values, the species and subspecies models performed generally better than the UTF and were more discriminative, respectively (Fig. 2). According to the calibration analyses, the occurrence probabilities of the species and subspecies models were under-fitted (slope: 1.31 and 1.44) and overestimated (bias: −0.58 and −0.43), respectively, while the predicted occurrence probabilities from the UTF are well calibrated (slope: 1.01 and bias: 1.44).

**Table 2:** The summarizing dimensions of the climate niche for each modeling subject using MaxEnt

Subject	Variable	I.(%)	Range	Curve
Subsp. *contorta*	Mean diurnal range	44.7	4–13	Unimodal, pos.
	Maximum temperature of dry quarter	29.8	−7–30	Unimodal, pos.
	Mean temperature of wet quarter	25.5	−7–19	Unimodal, pos.
Subsp. *murrayana*	Precipitation of warm quarter	40.3	1–232	Sigmoidal, neg.
	Precipitation of cold quarter	25.3	11–581	Sigmoidal, neg.
	Maximum temperature of dry quarter	14.9	−7–21	Unimodal, pos.
	Precipitation seasonality	9.8	19–110	Unimodal, pos.
	Precipitation of wet quarter	9.7	152–760	Sigmoidal, neg.
Subsp. *latifolia*	Mean annual temperature	72.8	−12–14	Unimodal, pos.
	Precipitation of dry quarter	14.7	0–262	Unimodal, pos.
	Precipitation seasonality	12.5	6–70	Sigmoidal, neg.
Species model	Mean temperature of warm quarter	72.8	0–22	Unimodal, pos.
	Precipitation of dry quarter	19.8	2–470	Unimodal, pos.
	Precipitation seasonality	7.4	20–75	Sigmoidal, neg.

A set of variables was found to be less correlated (*ρ <* 0.7) and important to explain the distributions of each subspecies. The ranges of variables (units are in Table 1), in which each subspecies responds to climatic conditions, and the shape of their response curve highlight the different characteristics of each subspecies, and provide an informative contrast to the conventional method (species model). Furthermore, the importance (I. (%)) of each variable, measured by the relative change in AUC when singularly randomizing, hints at the dominant climate factors depicted by each model.

It is notable that the species model predicts low occurrence probabilities of subspecies *murrayana* in the Sierra Nevada. All models predict a medium to high occurrence probability in eastern Canada and southwest Alaska where it is absent. The MaxEnt species model predicts a high occurrence probability in the Northwest Territories, where *P. contorta* is currently extending its range, but not to the extent predicted.

### Importance of intraspecific variability for future projections

**Figure 3:**
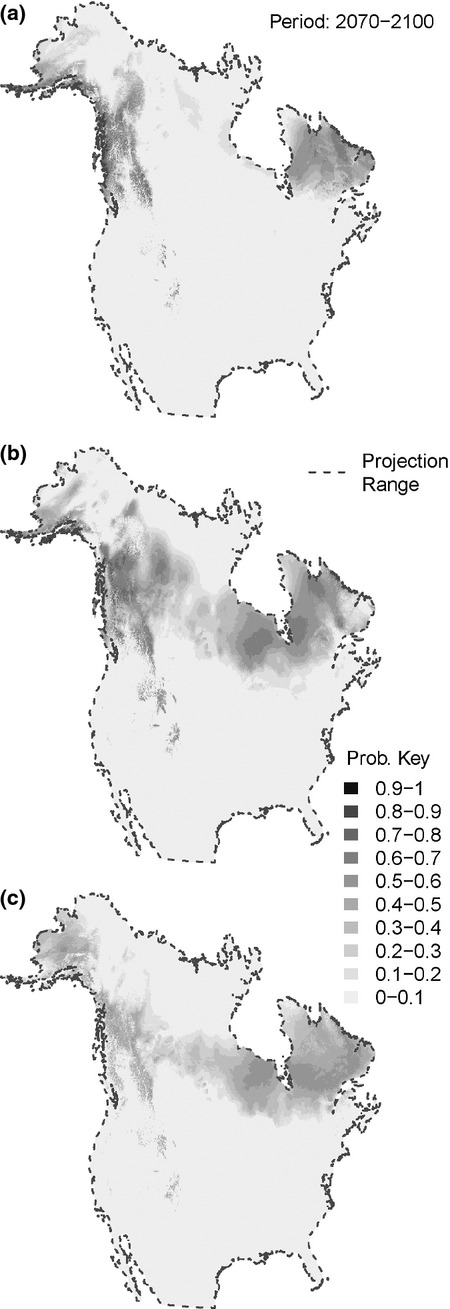
Projections of the three models (a) species model (b) subspecies model and (c) universal transfer function onto North American climate for the period 2070–2100 under the A2a emissions scenario from the HadCM3 global climate model.

**Figure 4:**
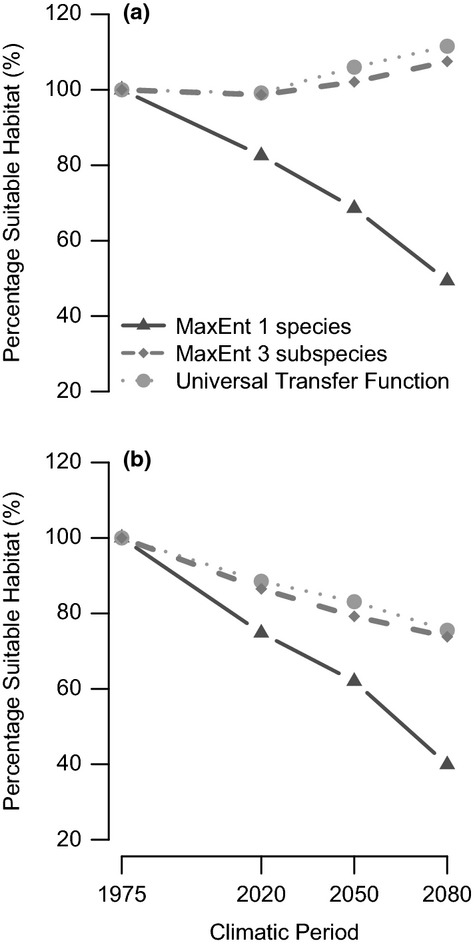
Predicted suitable habitat area of *Pinus contorta* relative to the reference climatic period 1950–2000 assuming (a) full dispersal and (b) no dispersal for each climatic period for all modeling techniques. Modeling techniques which incorporate intraspecific variability – the UTF and subspecies model, predict more optimistic outcomes for *Pinus contorta*.

**Figure 5:**
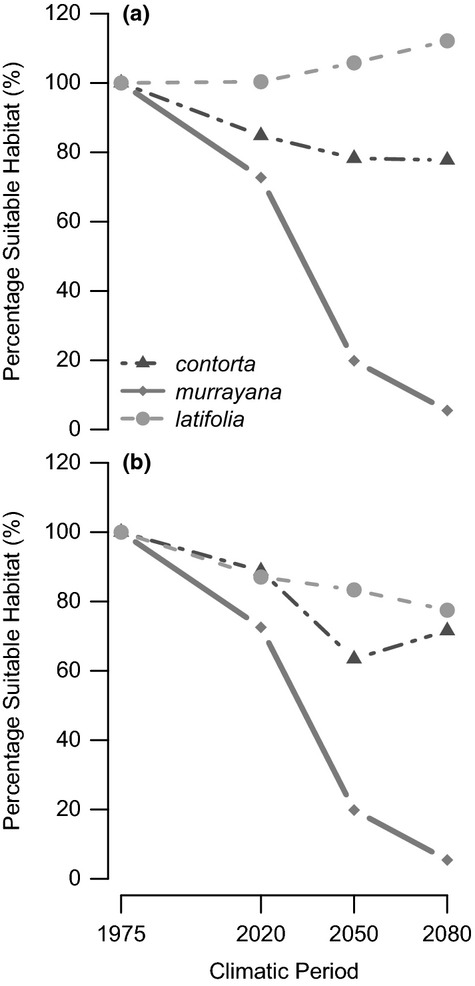
Relative predicted suitable habitat area of *Pinus contorta* subspecies assuming (a) full dispersal and (b) no dispersal for each climatic period (taken from the subspecies model).

Predicted future habitat suitability shows a general northward shift of suitable habitat (compare Figs. 3). The models predict different future development of suitable area, with a marked difference between the UTF or the subspecies model and the species model (Fig. 4); that is, the difference is between the models that do or do not incorporate intraspecific variability. More specifically, in a no-dispersal scenario, the UTF predicts 24% habitat loss for the period of 2070–2100 relative to the current scenario (1950–2000), and similarly the subspecies model predicts 26% habitat loss, while the species model predicts 60% habitat loss. Furthermore, given perfect dispersal to newly suitable climatic areas for the period of 2070–2100, the UTF predicts 12% habitat gain, and the subspecies model similarly predicts 8% habitat gain, but the species model predicts 51% habitat loss. As can be seen in Fig. 4, the predicted change in climatically suitable area is gradual over time. Examining the subspecies model projections more carefully (Fig. 5), predicted climatic change affects the subspecies differently: in a no-dispersal scenario, subspecies *latifolia* may lose current habitat (23% habitat loss) by the period 2070–2100, and subspecies *contorta* is also predicted to experience habitat loss (28% habitat loss), whereas the subspecies *murrayana* is predicted to drastically lose current habitat suitability (95% habitat loss). In a full dispersal scenario, subspecies *latifolia* may experience habitat gain (12% habitat gain), whereas subspecies *contorta* is predicted to experience a decrease in habitat suitability (95% habitat loss) as well as subspecies *murrayana* (95% habitat loss).

## Discussion

The three models exhibited similar performance and performed well for current distributions and climates. The species model was more discriminative than the UTF, but was under-fitted and yielded overestimated occurrence probabilities similar to the subspecies model. The UTF was less discriminative but better calibrated than either the species or subspecies models, whereas the species and subspecies models were more discriminative, but were poorly calibrated. The UTF approach used the occurrence data only via logistic regression to convert the predicted tree growth to occurrence probabilities; independently, the growth model was derived from the Illingworth provenance trial data. While the UTF approach still discriminates well, we see a non-trivial performance difference between the species or subspecies models, and the UTF. Notably, the subspecies model slightly outperformed the species model, coinciding with the results from Pearman et al. (2010).

The SDM model algorithm (e.g., Random Forest, GAM, MaxEnt, etc.) has been shown to be an important source of uncertainty (Dormann et al. 2008). However, in this study, we observed that incorporating intraspecific variation is another important source of variation in addition to model algorithm. Results of the species model and the other two models differed greatly in terms of predicted changes in the extent of future suitable habitat. In contrast, we did not observe a notable difference between the approaches driven by intraspecific data, that is, the subspecies model and the UTF (Fig. 4). In the two dispersal scenarios, the intraspecific approaches agreed with each other, but differed from the conventional species model, which predicted much greater climate change effects. Given that the two intraspecific approaches come to similar conclusions concerning climate change impacts suggests that the result does not depend on the particular way in which intraspecific variation is taken into account, and thereby corroborates previous research using the subspecies approach (e.g. Pearman et al. 2010). Results of our approaches including intraspecific information concur closely with predictions of net change in the extent of *P. contorta* climatically suitable habitat within BC from Hamann and Wang (2006) as well as Gray and Hamann (2012), whose community modeling approach yielded promising results for projecting ecolimatic shifts in forest stands (Gray et al. 2011; Gray and Hamann 2012), although they rely on the assumption that community composition and thereby ecological structure will endure. Gray and Hamann (2011) found that if *P. contorta* populations were unable to migrate, suitable habitat within seed zones would decline by 14% (2020s), 22% (2050s) or 34% (2080s), levels comparable to the “no dispersal” scenario of our intraspecific models (Fig. 4b). The species models from McKenney et al. (2007) predict less habitat change than our species model, whereas Coops and Waring (2010) using a process-based model predict a similar change in habitat change; nonetheless, the approaches utilizing intraspecific information presented here predict much lesser climate change impacts, given both perfect and no migration. This further indicates that migration abilities should be investigated at sub-species level.

Benito Garzón et al. (2011) came to very similar conclusions regarding the meaning of intraspecific information for *Pinus sylvestris* and *Pinus pinaster* in light of climate change using provenance trial survival data. Our study complements and corroborates their results with datasets that are more expansive and representative of the species subject, and focus on the growth response rather than mortality. In the case of *Pinus sylvestris*, the Eurasian analog to *P. contorta*, the range of the species that Benito Garzón et al. (2011) sampled represents a relatively small portion of species range, and the performance of their conventional SDM may be a result of the small geographic range of the occurrence data; Thuiller et al. (2004) present results demonstrating that the smaller the range, the more likely the outlook produced by SDMs will be grim. Although tree mortality is directly related to species distributions, tree mortality is difficult to predict (van Mantgem et al., 2009), as is also apparent in the low-explained survival variance presented by Benito Garzón et al. (2011), and influenced by a range of factors in addition to climate, that is, bark beetles, over-browsing, and other more proximal influences (Austin 2007). The convergent results of the study by Benito Garzón et al. (2011) and ours indicate the robustness of the underlying phenomenon and highlight the utility of provenance trials to investigate sub-species variation.

Regarding the use of conventional SDMs, the most cost- and time-efficient method to incorporate intraspecific variability is to model sub-specific variants individually and combine the respective projections afterward, which is presented first in Pearman et al. (2010) and again in this study. This approach is of course less informative than carrying out provenance trials, but serves the purpose of creating more realistic species distributions than conventional SDMs. Given the similarity of the methods presented here that incorporate intraspecific variability (subspecies model vs. UTF), it appears that “sub-clade” (Pearman et al. 2010) or subspecies models are a more robust approach to using occurrence data. As Pearman et al. (2010) noted, the less prevalent conspecifics are improperly represented in species (clade) models. Similarly, although not quantitated, we find that the species model underpredicts distributions of subspecies *murrayana* in the Sierra Nevada as well as subspecies *contorta* along the coast, where they currently thrive, which is not the case in the subspecies model (Fig. 5). The niche of the most prevalent subspecies *latifolia* appears to be best represented in the species model. Pearman et al. (2010) described prerequisites for dividing a species or taxon into subcomponents, which summarize to ecogeographical distinction of within-taxon ecotypes, in other words, substantial niche differentiation which is spatially segregated. Given the differences among the subspecies of *P. contorta*, the effects of climate may reasonably have a different effect on each subspecies and our results support this notion (Fig. 5). Pearman et al. (2010) show that 7 of 10 species, which exhibit considerable intraspecific variation, have greater projected range extents in future climates. Furthermore, their models, which incorporate within-species variation, outperform their species models, similar to our results (Fig. 2 and see Supporting Information). Species with much intraspecific variability such as *P. contorta* exhibit niche diversity and breadth, which is difficult to capture in a single model. In the case of *P. contorta*, it appears that the niche breadth was underestimated by the species model. All models predict suitability in Eastern Canada and Southwest Alaska, where *P. contorta* occurs rarely or is absent, suggesting that substantial niche overlap with other boreal tree species. As thoroughly discussed in the field of SDM (e.g., Guisan and Zimmermann 2000), only the realized niche is captured by SDM approaches. Perhaps *P. contorta* could occur more frequently in many other parts of Canada and in southern Alaska, but natural history, processes such as dispersal and competition, and/or forestry management have shaped current observation patterns. Furthermore, difference in realized and fundamental niches may also reflect evolutionary lag following the last glaciation or recent climate change (O'Neill et al. 2008). Given the range of climates that *P. contorta* inhabits, it is very possible that the predicted suitable areas would host *P. contorta* well.

The work of Thomas et al. (2004) initiated a wide discussion regarding the extinction risks associated with global climate change. In reply, Harte et al. (2004) argued that the conclusions therein may be conservative in that the averaging characteristic of SDM implies that one ecotype is as capable as another ecotype, from a climatically different portion of the species' range, of filling the modeled niche. Results from the Illingworth trial (Rehfeldt et al. 1999; Rehfeldt and Wykoff 2001; O'Neill et al. 2008) and other large provenance trials (Reich and Oleksyn 2008) concur in that tree growth decreases with increasing climatic distance between test site and population origin. However, when considering the UTF (i.e., O'Neill et al. 2008) across all provenances, the contribution of intraspecific variation appears to buffer climate change impacts. Thuiller et al. (2004) present results suggesting that species exhibiting greater niche variation have a greater chance of coping with climate change. Furthermore, results from this study (as well as Pearman et al. 2010) indicate that conventional species distribution modeling techniques are unable to properly estimate the niche breadth of intraspecific variants, especially if the intraspecific variants are differently prevalent, and may therefore result in future projections that overestimate suitability distribution losses (e.g., Thomas et al. 2004; Thuiller et al. 2008; Urban et al. 2012). Considerable intraspecific variability is a common feature of plant species with broad distributions as indicated by local adaptations (e.g., Joshi et al. 2001; Hufford and Mazer 2003; Savolainen et al. 2007; Kreyling et al. 2012b). We find that the individual subspecies models show the same or better performance than the single species model (Fig. 4), in accordance to the results of Kadmon et al. (2003). However, the performance of the combined subspecies model cannot easily be explained by the often better performance of models for species with smaller niches, because the combined model does not necessarily represent small niches, but rather a wider range of niches, and it is therefore relevant that the overall performance of the combined model was not lower than that of the single species model. Species with restricted distributions are on average more vulnerable to climate change (Thuiller et al. 2004; Schwartz et al. 2006), and species with little intraspecific variability or niche breadth are also most likely to be disadvantaged. While this study addressed intraspecific variation, all approaches assumed that the derived relationships to the environment do not evolve, as they likely would for real populations (Benito Garzón et al. 2011). However, our findings indicate that intraspecific variation alone already allows for buffering against environmental change. This is relevant as examples of niche conservatism, that is, the static nature of a niche, occur frequently in the fossil record (Davis and Shaw 2001) and in current studies (Wiens et al. 2009). Arguably, O'Neill et al. (2008) provide a compelling example of niche conservatism in *P. contorta*: the best predictor of transplanted tree growth was the mean cold monthly temperature of its *provenance* climate.

In conclusion, this study emphasizes the need to investigate intraspecific variation, if applicable, when considering assisted migration (Aitken et al. 2008; Kreyling et al. 2011). Whether using provenance trial data (e.g. O'Neill et al. 2008; Benito Garzón et al. 2011) or acknowledging ecogeographically unique intraspecific variants with a conventional SDM technique (e.g., Pearman et al. 2010), intraspecific information is a necessary addition to sapient SDM analyses. Our results show that incorporation of intraspecific variation results in very different, in this case much more positive, projections of future suitable habitat for a highly variable species. Assessing the extent to which species are able to utilize intraspecific variation presents a pertinent research challenge.

## Supporting Information

**Table S1.** Observation data were gathered from many sources. The largest sources of data are listed first. Distributional data (Figure 1) were obtained from many sources, including government agencies, but also from online resources such as herbaria, botanical gardens, and plant databases.

**Table S2.** Prevalence data that was presented to the Max-Ent models for each modeling target. The sum of subspecies occurrences do not add to the amount of occurrences for the entire species, because the subspecies co-occur in several locations, which is counted as a single occurrence for the whole species.

**Figure S1.** *Pinus contorta* subspecies distributions were given a 1,000km buffer and were truncated in the areas where no survey data exists. The smaller (truncated) areas refer to the model-building or the training area. The models built were then projected to the full subspecies range i.e. entire 1000km buffer called “subsp. projection range”. Each subspecies has its own modeling and projection range (see text). The modeling range represents the areas, where presence-pseudoabsence data is available.

**Figure S2.** *Pinus contorta* subspecies distributions were projected for the current period of 1950–2000 and then combined afterwards (Equation 1). Subspecies' *contorta* a) *murryana* b) and c) *latifolia* were modeled within their habitats with a 1000km buffer and then projected to the North American continent.

**Figure S3.** *Pinus contorta* subspecies distributions were predicted for the future period of 2070–2100 and then combined afterwards (Equation 1). Subspecies *contorta* a) is predicted to shift up along coastal areas, whereas subsp. *murrayana* may lose a substantial amount of habitat, and *latifolia* should show a dramatic shift northwards.

**Source code S1.** The R and GRASS code used to prepare, analyze and visualize the data in this study. Documentation is within the code as well as acknowledgements of the author(s) of the code.
