# Supplementary material for: Intraspecific variation buffers projected climate change impacts on Pinus contorta
Source: Ecol Evol. 2013 Jan 17;3(2):437–49. doi: 10.1002/ece3.426 (PMC3586652; doi:10.1002/ece3.426)
Supplement: Supplementary file 1 [file ece30003-0437-SD1.pdf]

Brian Oney, Björn Reineking, Gregory A. O'Neill, Juergen Kreyling, 2013. Intraspecific variation buffers projected climate change impacts on Pinus contorta.

Occurrence Data Description

Table S1: Observation data were gathered from many sources. The largest sources of data are listed first. Distributional data (Figure 1) were obtained from many sources, including government agencies, but also from online resources such as herbaria, botanical gardens, and plant databases.

Source	No. of obs.	No. determ. to subspecies
BC Ministry of Forests	112326	141
USFS Forest Inventory and Analysis	18012	0
Oregon Flora Project	1591	572
Rocky Mountain Herbarium	1092	1092
CalFlora Checklist	591	218
Consortium of California Herbaria	147	134
Global Biodiversity Information Facility*	101	99
Alaska Museum of the North Database Arctos	30	3
Washington Flora Checklist	28	28
Digital Atlas of the Vascular Plants of Utah	25	0
University of Alberta Vascular Plant Herbarium	17	10
Missouri Botanical Garden, Tropicos Database	13	10
Colorado State Herbarium	10	10
Bruce Bennett	8	5
Total	133991	2322

*Note: The Global Biodiversity Information Facility consists solely of online flora checklists, herbaria and museums, which include: University of Oxford, UNIBIO, IBUNAM, The New York Botanical Garden, University of Arizona Herbarium, Royal Botanic Garden Edinburgh, Louisiana State University Herbarium, Utah Valley State College, University of Alabama Biodiversity and Systematics, University of Kansas Biodiversity Research Center, Marine pScience Institute - UC Santa Barbara, Finnish Museum of Natural History, Canadian Museum of Nature.

Prevalence Data

Table S2: Prevalence data that was presented to the MaxEnt models for each modeling target. The sum of subspecies occurrences do not add to the amount of occurrences for the entire species, because the subspecies co-occur in several locations, which is counted as a single occurrence for the whole species.

	Presences	Absences	Prevalence
subsp. contorta	2048	276413	0.007
subsp. murrayana	1449	227344	0.006
subsp. latifolia	42342	512139	0.083
All subsp.	45785	525225	0.087

Modeling Ranges

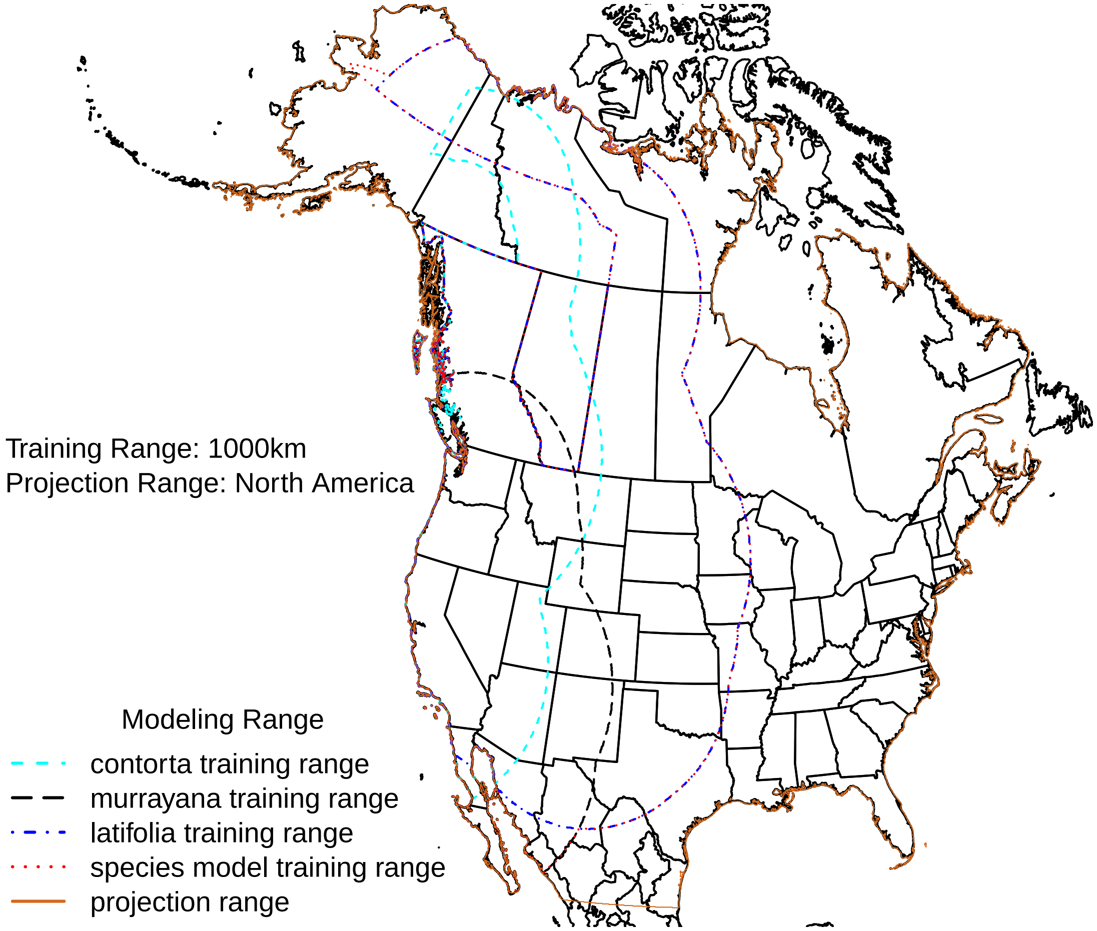

Figure S1: *Pinus contorta* subspecies distributions were given a 1,000km buffer and were truncated in the areas where no survey data exists. The smaller (truncated) areas refer to the model-building or the training area. The models built were then projected to the full subspecies range i.e. entire 1000km buffer called "subsp. projection range". Each subspecies has its own modeling and projection range (see text). The modeling range represents the areas, where presence-pseudoabsence data is available.

Subspecies Projections

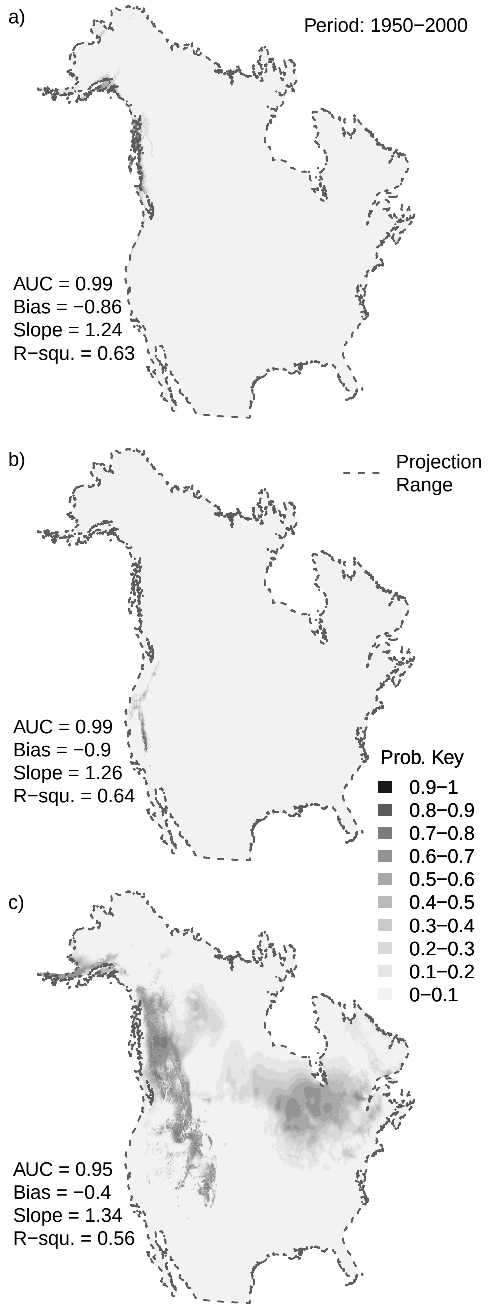

Figure S2: *Pinus contorta* subspecies distributions were projected for the current period of 1950–2000 and then combined afterwards (Equation 1). Subspecies' *contorta* a) *murryana* b) and c) *latifolia* were modeled within their habitats with a 1000km buffer and then projected to the North American continent.

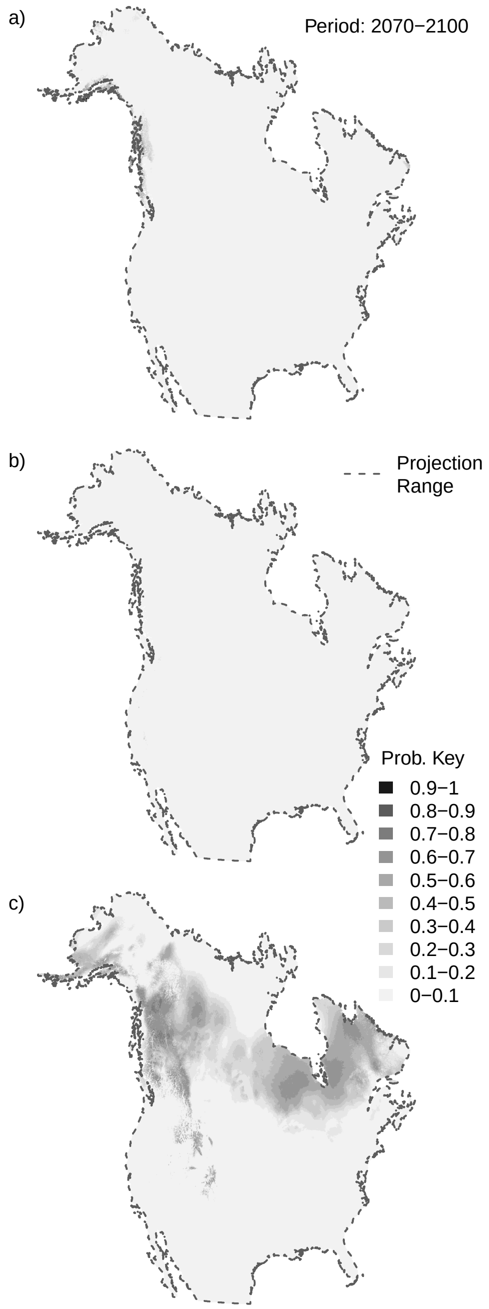

Figure S3: *Pinus contorta* subspecies distributions were predicted for the future period of 2070–2100 and then combined afterwards (Equation 1). Subspecies *contorta* a) is predicted to shift up along coastal areas, whereas subsp. *murrayana* may lose a substantial amount of habitat, and *latifolia* should show a dramatic shift northwards.
